# EBV-HSA-RegDB: A database of regulatory interactions between Epstein–Barr virus and human miRNAs

**DOI:** 10.1007/s00705-026-06753-8

**Published:** 2026-10-07

**Authors:** Helber Gonzales Almeida Palheta, Amanda Ferreira Mercês, Greice de Lemos Cardoso Costa, Gilderlanio Santana de Araújo

**Affiliations:** 1 Laboratório de Bioinformática e Ciências de Dados, Programa de Pós-graduação em Genética e Biologia Molecular, Instituto de Ciências Biológicas, Universidade Federal do Pará, Belém, Brazil; 2 Laboratório de Genética Humana e Médica, Instituto de Ciências Biológicas, Universidade Federal do Pará, Belém, Brazil; 3 Programa de Pós-graduação em Biologia de Agentes Infecciosos e Parasitários, Instituto de Ciências Biológicas, Universidade Federal do Pará, Belém, Brazil

## Abstract

Epstein-Barr virus (EBV) is a ubiquitous human herpesvirus associated with various malignancies and autoimmune diseases. MiRNAs play crucial roles in viral-host interactions by regulating gene expression post-transcriptionally. Understanding the regulatory networks between EBV-encoded miRNAs and human miRNAs is essential for elucidating viral pathogenesis and identifying therapeutic targets. We reported the EBV-HSA miRNA Regulatory Database (EBV-HSA-RegDB, https://lbcd.ufpa.br/ebvhsa or https://lbcd.ia.br/ebvhsa), an integrated bioinformatics resource that combines comparative sequence analysis, regulatory network reconstruction, experimental data integration, and functional gene enrichment analysis. The database incorporates miRNA sequences from miRBase, experimentally validated miRNA-target interactions from miRTarBase, transcriptomic data from cell line models, and miRNA data from ENCORI/StarBase. We implemented multiple sequence-comparison methods, including Hamming, Levenshtein similarity, and the Needleman-Wunsch alignment algorithm, to investigate miRNA seed and global alignments. The tool reconstructs and enables exploration of a comprehensive regulatory network linking experimental data on EBV-encoded miRNAs, human miRNAs, and their target genes. Gene Ontology and pathway analyses using the KEGG and Reactome databases are also available. Network analysis identified key regulatory hubs and revealed convergent targeting patterns in which EBV and human miRNAs regulate the same target genes (*IPO7*, *DICER*, *TP53*, *E2F3*, *SMAD4*, *SPRY2*) and several pairs of EBV-encoded miRNAs and their human counterparts, with high similarity in their target sequences. Functional enrichment analysis uncovered significant associations with biological processes, molecular functions, cellular components, and pathways relevant to viral infection, immune response, and cellular transformation. The EBV-HSA-RegDB provides a user-friendly, interactive platform for exploring virus-host miRNA regulatory networks. This resource facilitates hypothesis generation regarding EBV pathogenesis mechanisms and may aid in elucidating novel gene targets for EBV-associated diseases.

**Supplementary Information:**

The online version contains supplementary material available at https://doi.org/10.1007/s00705-026-06753-8.

## Introduction

Epstein-Barr Virus (EBV) is a human herpesvirus that encodes miRNAs (EBV-miRNAs), the first miRNAs identified in viruses [18]. These miRNAs play significant roles in various biological processes, particularly immune regulation and oncogenesis. The interaction between EBV-miRNAs and human miRNAs has become an area of increasing interest, particularly regarding their roles in tumorigenesis and immune evasion. Some studies show that more than half of the proteins encoded by the EBV genome are involved in this evasion, modulating the host’s immune response [21, 26].

Viruses evade host immune responses through EBV-miRNAs, which also contribute to oncogenesis. For example, they can modulate gene expression involved in apoptosis, proliferation, and immune signaling. EBV-miRNAs, such as those from the BART and BHRF1 clusters, have been shown to target host genes associated with tumorigenesis, thereby promoting the survival and proliferation of infected cells [7, 8, 12, 22, 26, 27, 32]. This targeting can redirect the host epigenome, favoring tumor development, particularly in cancers such as nasopharyngeal carcinoma (NPC) [3, 20, 23, 29]. This emerging evidence suggests that viral miRNAs can exhibit sequence similarity to host miRNAs, potentially allowing them to functionally mimic host regulatory molecules to hijack cellular regulatory networks, manipulate host cell biology, and evade immune surveillance.

Most computational resources, such as databases, have addressed aspects of viral miRNA biology. For instance, VIRmiRNA provides curated information on experimentally validated viral miRNAs and their targets, including EBV-miRNAs [19]. The miRBase offers detailed sequence and annotation data for EBV-encoded miRNAs. The VirusMINT focuses on host–virus protein interactions relevant to EBV pathogenesis and immune evasion [2]. The VISDB is an active comprehensive database of viral integration sites, emphasizing viral genomics and host–pathogen interactions over miRNA-centric regulatory mechanisms [24]. In addition, ViRBase v3.0 advances the field by cataloging virus–host ncRNA interactions [4].

To our knowledge, these tools lack integrative analysis of host interactions and functional context, failing to place sequences within interaction networks or to address ncRNA-mediated regulation. This reveals a critical gap: the absence of an integrated EBV-focused miRNA resource that enables comprehensive analysis of sequence features, regulatory interactions, and biological impact. To address this gap, we developed the EBV-HSA miRNA Regulatory Database (hereafter, EBV-HSA-RegDB), an integrated bioinformatics resource that compiles comprehensive genomic sequence information on EBV and human miRNAs. The tool includes multi-method sequence similarity analyses (Hamming and Levenshtein similarity and Needleman-Wunsch alignments), integrates experimentally validated miRNA–target interactions, reconstructs and visualizes regulatory networks, and conducts functional enrichment analysis to identify associated biological pathways across several layers. The tool provides an interactive, user-friendly web interface to access raw data and networks.

## Materials and methods

### Data collection and organization

MiRNA sequences for EBV and Homo sapiens were obtained from miRBase (version 22.1), a comprehensive, curated database of published miRNA sequences and annotations [13]. Sequences were downloaded in multi-fasta format and filtered using organism-specific prefixes (*ebv-* and *hsa-*), yielding 44 EBV-miRNA sequences and 2,656 hsa-miRNA sequences.

### Experimental miRNA-Target Interactions

Experimental, miRNA–target gene interactions were obtained from curated and experimental studies, including:

- The miRTarBase (release 2025) [5], a manually curated database of experimentally validated miRNA–target interactions containing over 2.5 million entries supported by strong experimental evidence, including reporter (luciferase) assays, Western blot analysis, quantitative PCR (qPCR), CLIP-seq (crosslinking and immunoprecipitation followed by sequencing), and other functional validation methods. The miRTarBase stores 15 interactions involving EBV-miRNAs and 9479 interactions involving hsa-miRNAs and target human genes.
- The ENCORI/starBase programming interface regularly updates annotations of human listed in miRBase in real time [31]. ENCORI/starBase combines extensive analyses from thousands of CLIP-seq datasets to identify high-confidence RBP-binding sites/regions using rbsSeeker. It also includes tools like rriScan, TDMDScore, and RBP-MotifScan for systematic studies of RNA-RNA interactions, miRNA degradation, and RBP-binding motifs. Additionally, ENCORI incorporates Pan-Cancer datasets, shRNA-RBP screens, and degradome-seq analyses, and provides APIs and visualization tools to support comprehensive research into the regulatory mechanisms of ncRNAs, mRNAs, and RBPs. Human miRNAs that targets genes.
- Interaction data from Ungerleider et al., were also incorporated. To examine viral miRNA targeting, the authors used the high-resolution Cross-Linking, Ligation, and Sequencing of Hybrids experiments in cell models, focusing on how EBV miRNAs alter the tumor cell transcriptome in primary EBV-associated Burkitt’s Lymphomas (BLs) and Gastric Carcinomas (GCs) [25]. The raw transcriptome interaction data for the Akata and SNU719 models, representing BL and GC were integrated. For the BL SNU719 model, the set of interactions comprised 16,601 interactions between EBV and human genes and 26,885 hsa miRNAs and human genes. For the Akata GC model, 521 interactions were from EBV, and 4899 were for hsa miRNAs and human target genes.

### Reconstructing regulatory network architecture

The EBV regulatory network was modeled to include the following: (a) interactions between EBV-miRNAs and hsa-miRNAs derived from sequence similarity were based on Hamming distance for seeds (> 0.75); (b) interactions between EBV-miRNAs and their target genes were obtained from experimental cross-species interaction data from cell culture transcriptomes; and (c) interactions between hsa-miRNAs and their target genes were obtained from experimental data. The experimental edges corresponded to interactions in miRTarBase, ENCORI/starBase and CLASH experiments [5, 25, 31]. Network visualization was performed using PyVis (version 0.3.2), a Python library for interactive network rendering built on the Vis.js framework.

### Functional and pathway enrichment analysis of miRNA networks

Functional enrichment analysis was conducted programmatically using g: Profiler, a remote platform for functional profiling of gene lists (https://pypi.org/project/gprofiler-official/). The analysis incorporated multiple annotation sources to comprehensively characterize the biological relevance of the identified genes. The enrichment analysis was performed for Homo sapiens genes using the g: SCS (Set Counts and Sizes) statistical framework in g: Profiler to correct for multiple testing. Statistical significance was defined as an adjusted p-value < 0.05, ensuring robust control of false-positive enrichment. Gene Ontology (GO) annotations were evaluated across three domains: Biological Process (GO: BP), describing coordinated biological programs carried out through multiple molecular activities; Molecular Function (GO: MF), referring to elemental biochemical activities at the molecular level; and Cellular Component (GO: CC), indicating the subcellular locations or structural contexts in which gene products operate. In addition to GO terms, pathway-based enrichment was assessed using curated biological pathway databases. These included the Kyoto Encyclopedia of Genes and Genomes (KEGG), which provides representations of metabolic and signaling pathways [11], and Reactome, a manually curated resource of biological pathways and processes [10].

### Sequence analysis methods

Hamming similarity measures the number of positions at which corresponding nucleotides differ between two sequences of equal length. For miRNA pairs with identical lengths, the Hamming distance was calculated as:

$$\:H\left(x,y\right)=1-\:\frac{\sum\:_{i}\left({x}_{i\:}\ne\:\:{y}_{i\:}\right)\:}{L}$$

where x and y denote the miRNA sequences, and x_i_ and y_i_ represent the nucleotides at position *i* in each sequence, respectively. *L* denotes the length of the miRNA sequence. An assessment of the seed region was performed because seed complementarity represents the primary determinant of miRNA target recognition.

Levenshtein distance (edit distance) measures the minimum number of single-character edits (insertions, deletions, or substitutions) required to transform one sequence into another. Unlike Hamming distance, Levenshtein distance accommodates sequences of different lengths, making it suitable for comparing miRNAs with length variations. The Levenshtein distance was computed using dynamic programming with the following recurrence relation:

$$\:L\left(x,y\right)=\mathrm{min}\left\{\begin{array}{c}L(i-1,\:j)\:+\:1\\\:L(i,\:j-1)\:+\:1\\\:L(i-1,\:j-1)\:+\:cost\end{array}\right.$$

where cost = 0 if xi = yj, otherwise cost = 1. The Levenstein package is available for free at https://pypi.org/project/python-Levenshtein/.

The similarity indexes and global alignment were employed as complementary measures to refine similarity assessment and strengthen confidence in both whole-sequence and seed-region similarity, thereby reducing spurious matches. Importantly, these metrics were not used as independent predictors of regulatory interactions. Instead, they served as supportive evidence for candidate miRNA pairs, with seed-region similarity as the primary biological criterion for inferring shared target regulation. By integrating alignment and distance metrics, the framework prioritizes EBV–human miRNA pairs that exhibit both conserved seed regions and high overall sequence similarity, thereby increasing confidence in potential cross-species regulatory interactions.

## Results

### The EBV-HSA-RegDB

**Fig. 1:**
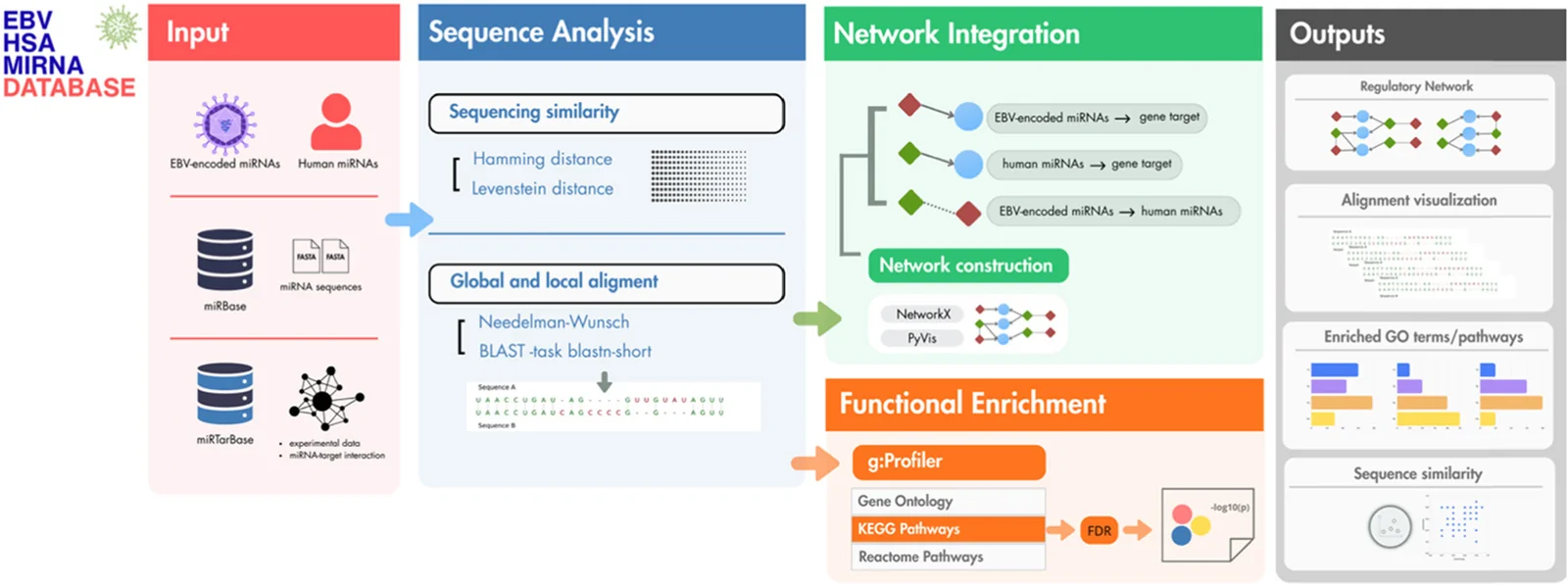
Overview of the EBV-HSA-RegDB workflow. The pipeline integrates Epstein–Barr virus (EBV)-encoded miRNAs and human (HSA) miRNAs as input, together with sequence repositories and experimentally validated interaction data. Sequence analysis includes similarity assessment using Hamming and Levenshtein distances, as well as global alignments using the Needleman–Wunsch algorithm. These data are integrated into regulatory networks linking EBV miRNAs, human miRNAs, and their gene targets. Functional enrichment analysis is conducted using g: Profiler, incorporating Gene Ontology, KEGG, and Reactome pathways, with multiple-testing correction (False Discovery Rate). The outputs include regulatory network visualizations, alignment plots, enriched biological pathways, and sequence similarity profiles, enabling systems-level interpretation of EBV–host miRNA interactions.=

The EBV-HSA-RegDB was implemented as a lightweight interactive web application using Streamlit (https://streamlit.io/) (version 1.28.0), a Python-based framework for developing data science applications. The EBV-HSA-RegDB supports automatic reactive view updates in response to user input and provides a comprehensive set of built-in widgets for interactive data exploration. An overview of the EBV-HSA-RegDB framework is shown in Fig. 1. The web application interface was organized into four main functional sections designed to simplify access to raw data, sequencing similarity and alignment, network integration, and pathway information.

- The Sequences section presents EBV-miRNAs and HSA-miRNAs sequences in a tabular format, along with associated sequence statistics, including identifiers, descriptions, and sequence lengths and external links for miRNA profiles. This module also enables users to export the displayed data in .csv format, supporting downstream analysis and reproducibility;
- The Hamming and Levenshtein analysis section displays precomputed distance metrics in a table and presents a scatter plot comparing Hamming and Levenshtein distances across miRNA pairs. Seed similarity was computed and shown for each pair of EBV and human miRNAs. Interactive hover tooltips reveal the identities of the corresponding miRNA pairs, enabling detailed, complementary cross-species comparative analysis.
- The Alignment section allows users to input pairs of miRNA sequences and graphically visualize their global sequence alignment using the Needleman–Wunsch algorithm.
- The Network Section comprises EBV-miRNA and HSA-miRNA interactions with target genes. The user can filter the experimental interaction data by a specific miRNA or by gene, to explore EBV miRNA evidence from miRTarBase, ENCORI/starBase, and CLASH experiments. Functional enrichment analyses are performed for each query and displayed for gene ontology and pathways. The Network section also displays a graphical network for EBV-miRNAs as green diamonds, HSA-miRNAs as red diamonds, and target genes as blue circles. Node sizes were scaled proportionally to node degree, reflecting the number of interactions, while edges were represented as lines connecting related nodes. Interactive features such as zooming, panning, hover tooltips, and node dragging were enabled to support exploratory analysis.

### The regulatory EBV-miRNA network and co-target interactions

#### Overview of experimental interactions

The reconstructed miRNA–mRNA regulatory networks exhibited substantial differences in size and composition. The SNU719 model network comprised 916 interactions among 32 EBV-encoded miRNAs, 69 human miRNAs, and 621 unique high-confidence target genes. The Akata model network displayed a comparable number of human miRNAs (*n* = 76), but contained fewer viral miRNAs (*n* = 20) and more target genes (*n* = 845) and regulatory interactions (*n* = 1,104). In contrast, the miRTarBase-derived network was the largest dataset analyzed, comprising 23,083 interactions among 975 miRNAs (10 EBV-encoded and 965 human) and 3,379 target genes. The markedly higher number of interactions reflects the comprehensive nature of miRTarBase, which integrates experimentally validated miRNA–mRNA interactions reported across multiple independent studies. Comparison of the three networks revealed that the cell line-specific models (SNU719 and Akata) contained a substantially higher representation of EBV-encoded miRNAs than the miRTarBase network, indicating greater coverage of viral regulatory interactions in these experimental contexts. Conversely, miRTarBase provided considerably broader coverage of human miRNAs and target genes, serving as a comprehensive reference for previously validated regulatory interactions.

#### The EBV miRNA regulatory network

**Fig. 2:**
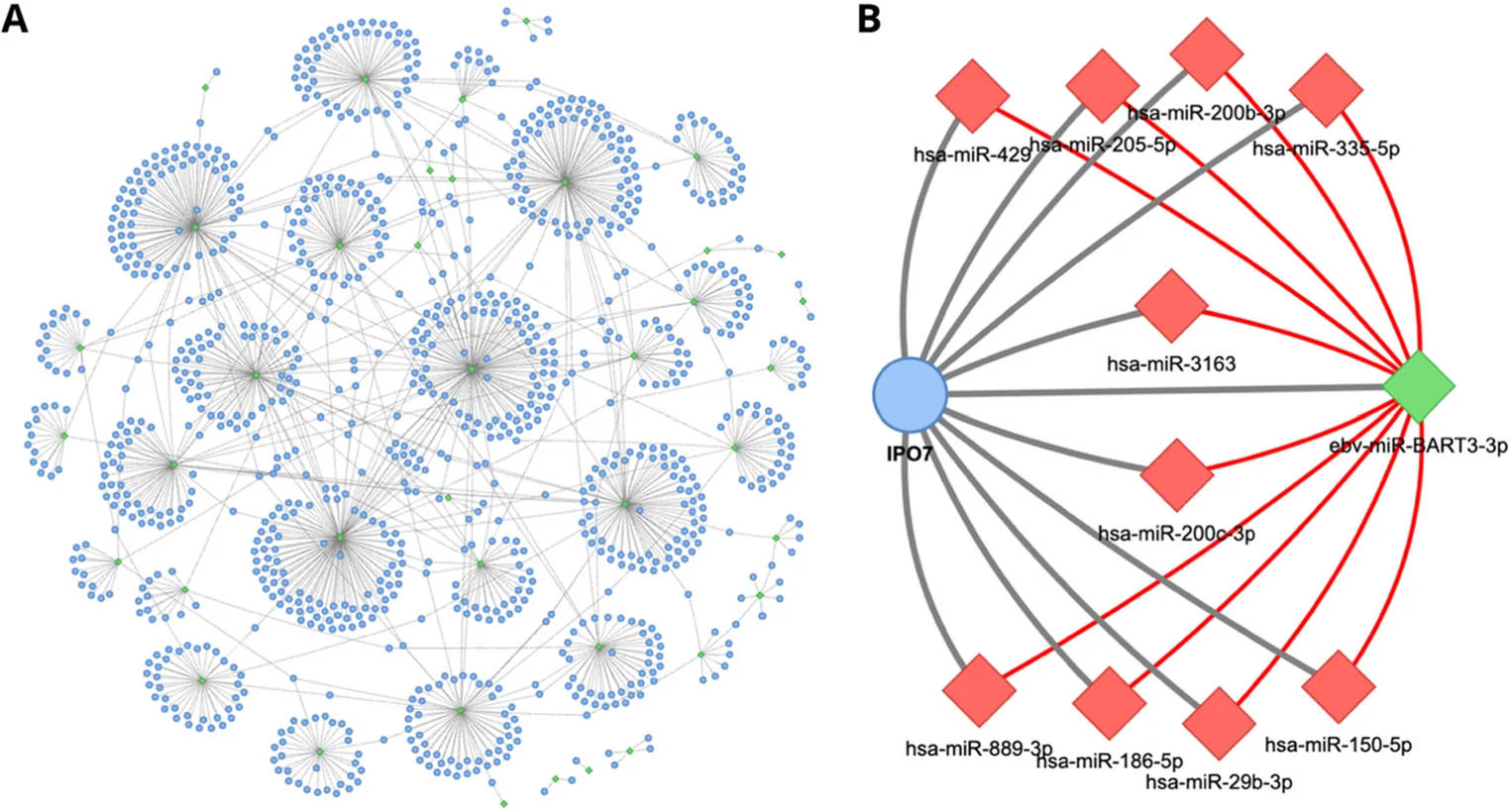
The reconstructed EBV miRNA–gene interaction network in EBV-HSA-RegDB. **A** The network was reconstructed from experimentally validated interactions in miRTarBase and from cell line-specific experiments (SNU719 and Akata). Dark green diamond-shaped nodes represent EBV miRNAs, whereas light blue circular nodes represent target genes. **B** The IPO7 gene network generated by the EBV-HSA-RegDB platform highlights experimentally validated interactions in miRTarBase and in cell line models, with Hamming similarity (≥ 0.75) between ebv-miR-BART3-3p and ten human miRNAs. The platform provides an interactive network visualization that allows users to inspect node attributes, such as node type and degree. Networks can also be downloaded in GraphML format for further analysis and customization in external software

We merged all interactions, including experimental studies, resulting in a complex regulatory network of EBV miRNAs and human genes (Fig. 2A). The network includes 434 nodes and 458 edges, with a low topological density of 0.005, typical for regulatory biological networks. It features 35 Epstein-Barr virus (EBV) miRNAs and 399 target genes, forming a structure where experimental interactions occur between miRNAs and their target genes. The network showed a hub-and-spoke layout, with multiple miRNAs converging on a few central genes. The connectivity is highly irregular, with a few viral miRNAs responsible for most interactions. Notably, ebv-miR-BART7-3p targets the highest number of human genes, with 150, followed by ebv-miR-BART5-5p with 76, ebv-miR-BART16 with 30, ebv-miR-BART9-3p with 25, and ebv-miR-BART17-5p with 22. Other EBV miRNAs have sets of targets ranging from 1 to 20 genes.

Analysis of gene connectivity identified *PAN3* as the most frequently targeted gene in the network, being regulated by four distinct EBV miRNAs (ebv-miR-BART9-3p, ebv-miR-BART8-3p, ebv-miR-BART5-5p, ebv-miR-BART1-3p). Eight additional genes were targeted by three EBV miRNAs (ebv-miR-BART7-3p, ebv-miR-BART16, ebv-miR-BART15), including *SOX4*, *SPTBN1*, *RHOC*, *KCMF1*, *CD47*, *ATP1A1*, *KRT8*, and *PANK3*. Furthermore, two EBV miRNAs targeted several genes, including *PPP1R15B*, *MAP7*, *WDR77*, *CSDE1*, *LARP1*, *LGR4*, *RAB13*, *UBE2H*, and *CD74*.

Figure 2B depicts the IPO7 regulatory network generated using the EBV-HSA-RegDB platform, which highlights experimental interactions of ten human miRNAs with high Hamming similarity (≥ 0.75) to the viral miRNA ebv-miR-BART3-3p. The network integrated experimentally validated interactions with sequence similarity analysis, highlighting potential regulatory relationships involving IPO7. The identified human miRNAs exhibited seed similarity to ebv-miR-BART3-3p, suggesting shared target recognition patterns within the network.

Gene enrichment analysis of host genes targets of EBV-miRNAs showed significant overrepresentation of biological processes related to immune regulation and virus–host interaction (FDR < = 0.05) (Supplementary Table 1). Among the most significantly enriched Gene Ontology terms were negative regulation of immune response, negative regulation of innate immune response, regulation of alpha-beta T cell activation, antigen processing and presentation of peptide antigens, cellular defense response, and antimicrobial humoral immune response mediated by antimicrobial peptides. In addition, enrichment of the non-canonical NF-κB signaling pathway is consistent with EBV’s ability to regulate this axis, which plays important roles in immune modulation, cell survival, and viral persistence. The network was also enriched for cellular processes, including RNA modification, nucleotide biosynthetic processes, monocarboxylic acid transport, organic hydroxy compound transport, detoxification, and regulation of the G2/M transition of the mitotic cell cycle, indicating potential involvement in metabolic adaptation and control of host cell proliferation. Other enriched terms, such as cartilage development, cilium movement, and adult behavior, likely reflect the broader functional diversity of genes represented in the network and their involvement in multiple physiological contexts.

Enrichment analysis of molecular functions within the EBV-associated network revealed significant enrichment of terms related to immune recognition, cytokine signaling, ion transport, and regulatory protein interactions (Supplementary Table 2). The most prominent enriched functions included immune receptor activity, antigen binding, peptide antigen binding, cytokine activity, cytokine binding, and cytokine receptor binding, indicating a strong association of the network with immune communication and host–pathogen recognition mechanisms. The enrichment of antigen-binding functions is consistent with the involvement of EBV-associated genes in immune surveillance and in the modulation of antigen presentation pathways. In addition, several ion channel-related functions were enriched, including monoatomic ion channel activity, gated channel activity, ligand-gated channel activity, and calcium ion transmembrane transporter activity, suggesting potential regulation of cellular signaling events mediated by ion fluxes. Other enriched molecular functions, such as enzyme–substrate adaptor activity and ubiquitin-like ligase–substrate adaptor activity, highlight the involvement of protein regulatory mechanisms, including post-translational modification and control of protein stability. The presence of serine hydrolase activity, lipase activity, and organic/carboxylic acid binding functions further suggests alterations in enzymatic and metabolic processes within the network. Additionally, enrichment of extracellular matrix structural constituent functions indicates potential roles in regulating cell–matrix interactions and tissue-associated responses.

## Discussions

In this work, we systematically curated EBV-miRNA sequences and co-target events using the human genome and experimental interaction data to propose the EBV-HSA-RegDB tool, aiming to fill gaps in pathogen-host interactions. The EBV-miRNA sequence similarity in the seed region of HSA-miRNAs primary supports the hypothesis of functional molecular mimicry, in which viral miRNAs can exploit host regulatory networks by converging shared or partially overlapping mRNA targets. Such mimicry could allow EBV to modulate essential host cellular pathways, including proliferation, apoptosis, and immune responses, through mechanisms analogous to regulation mediated by endogenous miRNAs. This indicates that the EBV can exploit post-transcriptional networks already established in the infected host cell. The hypothesis that EBV-miRNAs share sequence characteristics with specific human miRNAs, enabling convergent regulatory effects on common target genes, contributes to regulatory mimicry between host and virus. This strategy allows integration into the cellular regulatory network, favoring persistence and maintenance of viral latency.

The EBV-HSA-RegDB reveals biologically relevant interactions by integrating database records with experimental data from cell line models. The IPO7 regulatory network, created using the EBV-HSA-RegDB platform, combines validated human miRNA–target interactions with sequence similarity analysis to identify potential regulatory relationships with the viral miRNA ebv-miR-BART3-3p. It includes ten human miRNAs with high Hamming similarity scores (≥ 0.75) to ebv-miR-BART3-3p, indicating sequence conservation and potential target overlap. By merging experimental data and computational predictions, the network offers a comprehensive view of miRNA-mediated regulatory connections involving *IPO7*. These miRNAs share conserved seed regions or other features with ebv-miR-BART3-3p, suggesting shared regulatory mechanisms and convergence on host gene regulation. This approach characterizes host–viral miRNA relationships and identifies candidate regulatory circuits through which EBV-associated miRNAs may affect cellular pathways. The network highlights the complexity of post-transcriptional regulation involving *IPO7* and provides a framework for exploring interactions between viral and human miRNAs. Yang and Sugden report that *IPO7* is an essential host gene and that EBV miRNA–dependent regulation reduces IPO7 levels, conferring selective advantages on EBV-infected cells, supporting a model in which EBV actively modulates *IPO7* levels via viral miRNAs [28]. A related review of EBV miRNAs notes that BART family miRNAs (including BART3-3p and BART16) downregulate *IPO7*, linking this regulatory axis to altered cytokine production (e.g., *IL-6*) and to host-pathogen interactions during infection [15], and reiterates *IPO7* as a recurring EBV miRNA target.

Within the regulatory framework of EBV-HSA-RegDB, PAN3 is an important component linking miRNA recognition to post-transcriptional control of gene expression. During EBV infection, modulation of PAN3-containing deadenylation machinery may contribute to the regulation of host transcripts involved in viral replication, latency maintenance, and oncogenic processes. Therefore, alterations in the *PAN2*-PAN3 and CCR4-NOT pathways may represent a mechanism by which EBV reshapes host gene regulatory networks, promoting viral persistence and potentially contributing to EBV-associated disease progression (Frédérick & Simard, 2021; Kankanala & Manga, 2023; Lei et al., 2022).

Particularly, the potential interaction between EBV-miR-BHRF1-1 and the *TP53* gene warrants particular attention. The *TP53* gene encodes the p53 protein, a central transcription factor in maintaining genomic integrity, playing a role in inducing cell cycle stop, DNA repair, and apoptosis in response to stress or genetic damage. The modulation of this pathway by viral miRNAs represents a critical point of molecular interference, given p53’s role in regulating cell proliferation and preventing neoplastic transformation. Experimental evidence suggests that miR-BHRF1-1 can influence components of the p53-associated pathway in chronic lymphocytic leukemia cell lines, altering the balance between cell survival and death [16]. Similarly, the EBV-HSA-RegDB database highlights the interaction between EBV-miR-BART6-5p and the *SMAD4* gene, a central mediator of the TGF-β signaling pathway, which is widely recognized for its role in tumor suppression across various cancer types. This gene acts in the transduction of antiproliferative and pro-apoptotic signals and regulates the expression of genes involved in cell cycle control. The loss or functional reduction of *SMAD4* has been associated with tumor progression, increased invasiveness, and worse clinical outcomes in several neoplasms [30]. These findings support the presence of second-order regulatory mechanisms, in which EBV miRNAs influence not only specific downstream targets such as *TP53* or *SMAD4* but also upstream regulators that govern entire gene networks. This amplifies the biological significance of viral interference and underscores the complexity of virus–host interactions represented by EBV-HSA-RegDB.

Beyond well-characterized genes, the platform also enables the identification of less explored targets, such as *SPRY2* and *PDLIM7* that have shown possible interactions with EBV miRNA. Targetome studies of EBV-miRNAs identify these target genes as promising candidates for future investigations, possibly due to their roles in cell-signaling pathways involved in proliferative processes [6, 23]. The potential interaction of EBV-miR-BART6-5p with the *DICER1* gene has significant biological relevance. This gene encodes an endoribonuclease critical for the biogenesis of mature and functionally active miRNAs. Thus, its modulation can have amplified or diminished effects on the activity of this ribonuclease, compromising the maturation of tumor-suppressor miRNAs, destabilizing the balance between pro- and anti-proliferative miRNAs, and promoting extensive remodeling of the transcriptomic landscape [1, 7, 9, 14, 17, 29].

Consistent with this network-level perspective, additional targets identified in the database include *E2F3*, associated with proliferation control, and *STIM1*, associated with intracellular signaling. These findings reinforce the hypothesis that viral miRNAs act at multiple regulatory levels [23]. Instead of exclusively targeting isolated genes, these miRNAs may interfere with critical cellular networks, promoting functional reprogramming that favors both latency and, in certain contexts, tumor progression. This global miRNA imbalance has already been observed in several neoplasms associated with EBV infection, such as nasopharyngeal carcinoma, gastric cancer, and Hodgkin’s lymphoma [1, 7, 14, 17, 29].

Notably, sequence-based statistical analyses confirm that the interactions between EBV and human miRNAs are not mere random alignments of short sequences. Instead, these interactions are enriched for high sequence similarity and contain functionally significant seed regions. Consequently, although EBV-HSA-RegDB offers a thorough and integrated framework for generating hypotheses, additional studies and experimental evidence are necessary to prove causality and assess the extent of regulatory influence on co-target genes.

In conclusion, The EBV-HSA-RegDB provides a comprehensive, integrated resource for exploring Epstein-Barr virus-host miRNA regulatory networks. Through multi-method sequence analysis, network reconstruction, and functional enrichment, the database reveals: (a) sequence-level similarities between EBV-encoded miRNAs and human miRNAs suggesting potential molecular mimicry; (b) convergent targeting patterns where viral and host miRNAs regulate common genes; (c) functional enrichment in pathways critical for viral infection, immune response, and cellular transformation; and (d) network architecture highlighting regulatory co-targeting pathways. The interactive web interface enables researchers to explore these relationships, generate hypotheses, and identify candidates for experimental validation. As our understanding of virus-host interactions continues to evolve, resources like this database will play increasingly important roles in translating genomic information into therapeutic strategies. The EBV-HSA-RegDB is a valuable tool for the virology, immunology, and cancer research communities to dissect EBV pathogenesis and contribute to the development of novel diagnostic and therapeutic approaches for EBV-associated diseases.

## Supplementary Information

Below is the link to the electronic supplementary material.

Supplementary Material 1

## Data Availability

All data supporting the findings of this study are available in the paper and online in the EBV-HSA-RegDB , freely accessible at https://lbcd.ufpa.br/ebvhsa/ or https://lbcd.ia.br/ebvhsa. Raw sequence data were obtained from miRBase (https://www.mirbase.org/) and miRTarBase (https://mirtarbase.cuhk.edu.cn/) and ENCORI/StarBase via API (https://rnasysu.com/encori/).
